# Responding to old problems in the Dutch work addiction scale: a psychometric approach in a Peruvian sample

**DOI:** 10.1186/s40359-025-02437-1

**Published:** 2025-02-22

**Authors:** Edwin Salas-Blas, Miguel Vallejos-Flores, Gustavo Calderón-De la Cruz, Eduardo Manzanares-Medina, Eduardo Fonseca-Pedrero, Nikol Mayo-Puchoc, Anthony Copez-Lonzoy

**Affiliations:** 1 Universidad Peruana de Ciencias Aplicadas, Santiago de Surco, Peru; 2 Universidad Nacional Federico Villarreal, Lima, Peru; 3 Universidad de la Rioja, Logroño, Spain; 4 Universidad San Ignacio de Loyola, Lima, Peru; 5 Instituto Peruano de Orientación Psicológica, Lima, Peru

**Keywords:** Work addiction, Psychometric, Engagement, Exploratory graph analysis, unidimensionality

## Abstract

Harmful work patterns have become a public health problem. These conditions are worrying since they can have an impact on mental health, social conflicts, and physical difficulties due to their normalization of pathological behaviors related to work addiction. Aims: (1) determine the psychometric properties and reliability of the DUWAS and (2) evaluate valid comparisons at the construct level. Methods: The Spanish version of DUWAS was used. For the evaluation of the psychometric model, 446 Peruvian workers responded to the Spanish version (60.5% women aged 18 to 65, M = 35.1 SD = 12.45). Results: The unidimensional version was confirmed (*x*^2^ = 67.495_(14)_; CFI = 0.96; TLI = 0.95; SRMR = 0.050 and RMSEA = 0.069). The reliability was acceptable (α = 0.735; 95% CI: 0.706 − 0.762; ω = 0.741; 95% CI: 0.712 − 0.767) and measurement invariance was confirmed for sex and age groups. Concurrent validity was mostly identified with mental health indicators. Conclusion: This study provides new information on dimensionality and the realization of valid comparisons that allow its use in Spanish-speaking workers.

**Supplementary Information:**

The online version contains supplementary material available at 10.1186/s40359-025-02437-1.

## Highlights

The one-dimensional model is confirmed.

Work addiction is invariant across gender and age groups.

Indicators of mental health and personality were identified.

**Supplementary Information:**

The online version contains supplementary material available at 10.1186/s40359-025-02437-1.

## Background

Work addiction (WA) is a complex behavior with long-term negative consequences that include excessive, compulsive, and persistent work behavior, relational problems (family and friends), health problems, and deterioration of personal life [1, 2]. However, this addictive behavior should not be confused with workaholism even though they share common conditions of “hard work.“ [3, 4]. This difference lies in the ambivalent perception (positive or negative) about working for long hours with few or no consequences [1, 5] or from a positive analytical perspective, some consider that it has constructive and beneficial effects and that direct relationships have been found with job satisfaction and work productivity [6].

This addictive behavior may initially be related to positive reinforcements, such as promotions, representative trips, incentives, and raises [7], but then, this behavior would no longer occur to obtain the usual reward, but to avoid or escape from harmful stimuli [8, 9]. However, in official classification manuals such as DSMV (Diagnostic and Statistical Manual of Mental Disorders) and ICD11 [10, 11] Only two behavioral addictions are formally recognized: gambling and gaming disorders.

Despite this, reports on the prevalence of this phenomenon among workers vary between different cultures; in the Norwegian population it amounts to 8.3% [12] and 39.7% in South Koreans [13] and around 10% in the USA [14]; among different types of jobs held; among physicians who worked in the pandemic era up to 24.4% [15] and in nurses up to 28.3% [16, 17]; among university staff it was reported up to 35.5% [18–20]. Despite the growing interest in studying this phenomenon in different cultures and types of work, no information on this type of addictive behavior has been found in Latin America.

People with this type of problem behavior are characterized by being controlling and feeling the need to work during their breaks (e.g., weekends and/or vacations) [21, 22]. They even deny that their work behavior is responsible for their problems [9, 22]. This can generate a deterioration in the quality of life associated with work [21, 23]; the interaction of the person in their work environment, and a deficit of nuclear bonds generating a work-family conflict [24].

Recent studies have found evidence of increased WA in women [16] and among young adults aged 20–35 years [25]. Exposure to complex contexts (e.g. the COVID-19 pandemic) intensified remote work by increasing the time of undifferentiated work environments [26], this implies a continuous reinforcement in work activities out of time (e.g. non-working days and weekends), which may increase the “no risk perception” in the worker despite the lack of rest, generating stress and emotional fatigue, aspects that may favor this addictive behavior [27].

Literature about WA evidence involvement increment with general health problems (mental and physical) [2, 15, 18, 21, 28–36]. Mental health issues have been identified as negative affect, anxiety, chronic stress, burnout, mental distress, loneliness, depression, loss of enjoyment of leisure time, and aggressive behavior [2, 15, 18, 21, 28–31, 33, 34]. Also, relationships have been found with physical ailments, such as disabling low back pain, psychosomatic symptoms, and cardiovascular risk and fatigue [32, 34–36].

**Fig. 1:**
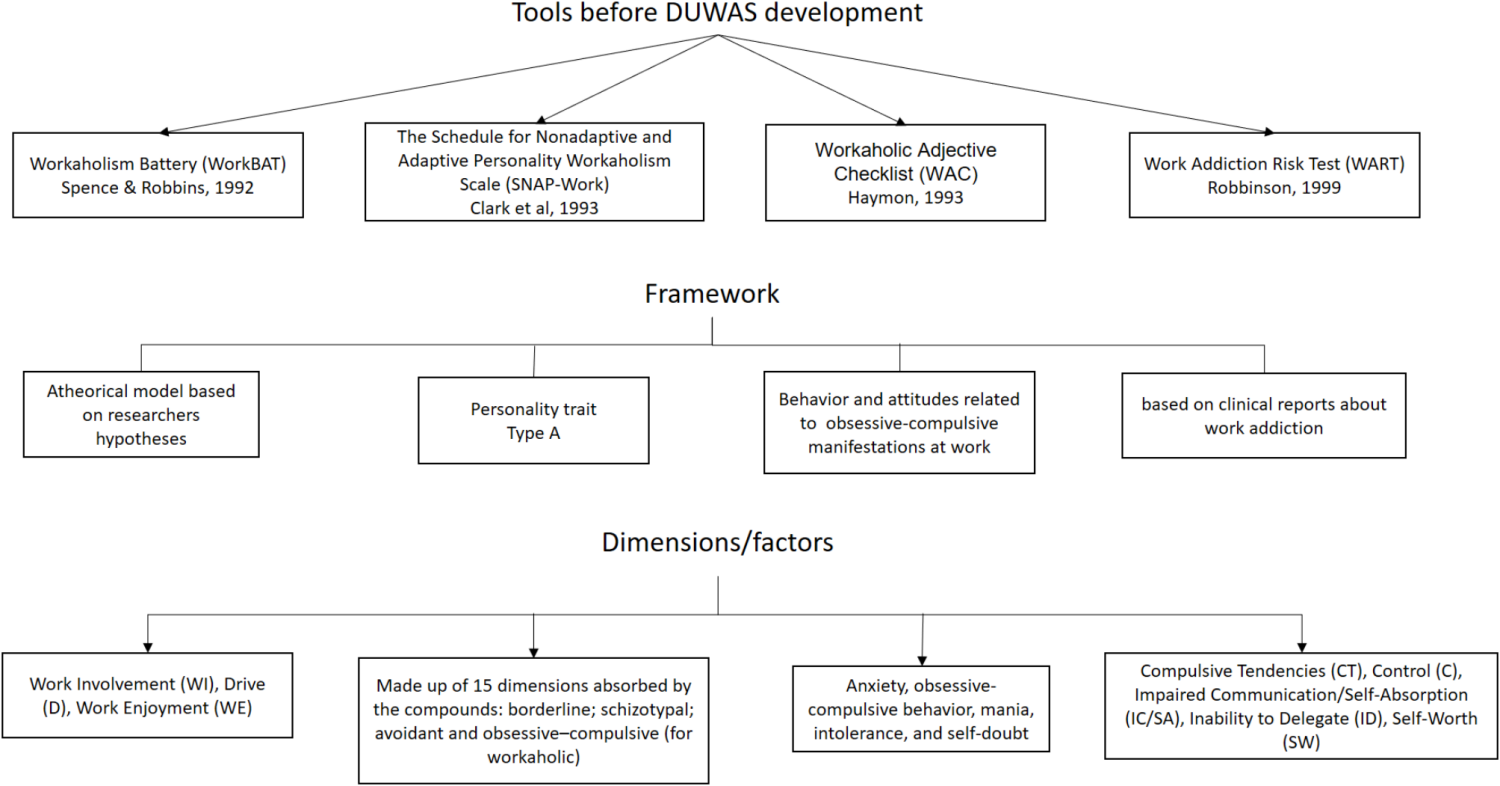
Initial tools to assess work addiction

Different questionnaires have been constructed to assess WA, such as the Bergen Work Addiction Scale [37, 38]; the Work Addiction Risk Test (WART) [39], the Workaholism Battery (WorkBAT) [40]. However, they do not all start from the same definition of WA, so it can be argued that the measures they perform are not comparable with each other (Fig. 1). The most widely used tool is the Dutch Work Addiction Scale (DUWAS), whose structure is supported by two dimensions such as working excessively (WE) and working compulsive (WC) [4, 41–50]. DUWAS has been evaluated in different contexts such as Europe [4, 43, 51], Asia [46, 52], Africa [53] and Latin America [42, 48, 54] with different results at a psychometric level (see supplementary material).

Within the literature about the DUWAS, some aspects deserve special review (a) problems directed to dimensionality, which does not allow a clear identification of the construct, biasing the results and possible interpretations of the construct. The heterogeneity evidenced in the DUWAS model includes several models such as three dimensions (incorporating Overwork items) [52], a unidimensional version that only includes the WE alone dimension as a central element in the process of WA [55] and, a second order factor for four subdimensions [53, 56]; (b) overlap between dimensions, overlap between WE and WC dimensions has been identified [4, 50, 53] which could indicate the experimentation of WA as a possible continuum rather than the objective difference of this addictive behavior, which may lead to the idea of ​​a possible unidimensionality; (c) overlapping items, a high correlation between previously identified pairs of short-form items centered on the WC dimension (e.g., pairs 3 and 7; 5 and 7 and 9 and 10) [4, 44, 45, 48] which would indicate an increase in information that is not relevant to users and could lead to misinterpretations (founded on an incorrect measurement model) that inflate the reliability scores leading to an overestimation of the instrument’s precision [4, 44, 45]; (d) In the absence of valid comparisons, measurement invariance allows determining that the measurement properties of an instrument do not change based on certain characteristics of the target groups. This has been a very little studied aspect for important differential groups that can identify a greater impact of this phenomenon (for example, gender and age groups) [25, 44].

DUWAS studies assessing concurrent validity with other WA-related instruments have identified direct relationships with overtime, overwork (convergent), and burnout syndrome [52]. Inverse relationships with engagement (discriminant) were also identified [57]. In addition, WA has been related to enjoyment and absorption at work [58], finding that some addicts could have high scores of absorption and enjoyment, due to the dedication that addicts have with work, although the motivations for this dedication are influenced by this addictive behavior [59]. Regarding reliability, internal consistency was evaluated in most cases with Cronbach’s alpha, reporting acceptable values above.73. Mainly the overwork dimension has a higher identifiable accuracy [4, 53, 56, 60]. However, the accuracy of DUWAS scores only remains stable with “large data sets [4, 43, 44, 56, 60].

Despite the growing interest in studying this phenomenon in different regions, cultures, and types of work [61], insufficient information has been found in Latin America. The aims of the study were (1) to determine the internal structure and reliability of the DUWAS in the Spanish version, (2) to analyze the invariance of measurement between groups by sex and age, and (3) to determine the concurrent validity between variables related to positive work engagement.

## Methods

### Study design

The study design was a cross-sectional investigation.

### Participants

The dataset was analyzed with the inclusion criteria: (1) 18 to 50 years of age (2) must be currently employed (at least 6 months) (3) participants had complete data on DUWAS, PHQ-9, GAD-7, and BSSS scores (4) Participants had to have agreed to participate in the study first after providing informed consent. We excluded participants with implausible data (i.e. age > 99 years). The evaluation of the psychometric properties of DUWAS was carried out on young adults and Peruvian adults who were currently working in Lima, the most populated capital of Peru. People who only worked part-time or with contracts of less than six months were excluded. Non-probabilistic snowball sampling was carried out. To mitigate possible sources of error (e.g. problems in the meaning or phrasing of the items), an initial pilot study of 30 participants was carried out. This group did not report difficulties, so the application was continued in the target sample. Participation was free and voluntary without any type of financial compensation. Recruitment and administration of participants were done through social networking sites (e.g. Facebook). Initially, target participants (who met the enrollment criteria) were considered. To increase the dissemination of the survey, two routes were taken: (a) the creation of Facebook groups, where the referrals reposted the survey link and (b) sending via FB messenger (hosting the survey link). Missing data were not found. The sample size calculation suggests values lower than *n* = 300. For the present study, 466 Peruvian youth and adults were used.

### Instruments

#### Dutch work addiction scale (DUWAS)

DUWAS [60], is a self-report scale developed to assess WA. The short 10-item version assessing the dimensions of Working Excessively (WE, 5 items) and Working Compulsively (WC, 5 items) was used. The response range is ordinal and varies from 1 (“almost never”) to 4 (“almost always”). In the original version, the DUWAS reported variant validity evidence at the internal structure level (RMSEA = 0.08, TLI = 0.90, CFI = 0.93). The reliability of the DUWAS values had heterogeneous results for both dimensions, for example, WE (α = 0.69 to 0.88) and WC (α = 0.64 to.83).

#### Utrecht work engagement scale (UWES)

The 9-item version of the Utrecht work engagement scale (UWES) consisting of the dimensions of (1) Vigor (items 1, 2 and 5); (2) Dedication (items 3, 4 and 7), and (3) Absorption (items 6, 8 and 9) [62]. The items are scored on a 7-point Likert scale, from 0 = “never” to 6 = “always”. Peruvian validation was considered [63], confirmatory factor analysis reported satisfactory fit indices: CFI = 0.99, RMSEA = 0.000 (CI90%= 0.000, 0.045), factor loadings were greater than 0.669, interfactor relationships were between 0.474 and 0.882. The internal consistency was performed with Cronbach’s alpha coefficient (α) obtaining the following values: Vigor 0.77 (IC95%= 0.69 to 0.82), Dedication of 0.74 (IC95%= 0.65 to 0.80) and Absorption 0.61 (IC95%= 0.49 to 0.70). These variables are important because they assess work-directed behavior and possible maintenance conditions.

Patient Health Questionnaire − 9 (PHQ-9) is a self-reported screening tool for symptoms of major depression. The score is obtained summatively, ranging from 0 to 27 points with Likert-type responses (0 = not at all; 1 = several days; 2 = more than half of the days; 3 = almost every day) [64, 65]. In Peru, PHQ-9 has demonstrated adequate reliability (ω = 0.87) and optimal fit for the unidimensional model (CFI = 0.936; RMSEA = 0.089 and SRMR = 0.039) [66].

The Generalized Anxiety Disorder-7 (GAD-7) is a brief screening scale consisting of 7 items that assess generalized anxiety disorder [65]. Response options range from 4 points (0 = none; 1 = several days; 2 = more than half the days; 3 = almost every day). The GAD-7 model has identified a good fit (CFI = 0.977; TLI = 0.966; SRMR = 0.043; RMSEA = 0.076) and reliability (α and ω > 0.70) [67].

Brief Sensation Seeking scale (BSSS) is measured and consists of 8 items [68, 69]. Responses are evaluated in a Likert-type format ranging from “strongly disagree,” “disagree,” “neither disagree nor agree,” “agree,” and “strongly agree.” The BSSS-8 has acceptable reliability (ω = 0.74) and an optimal fit of its internal structure (CFI = 0.977; TLI = 0.941; SRMR = 0.036; RMSEA = 0.047) [70].

### Procedures

#### Item analysis

We described the distribution of the DUWAS items reporting their mean, standard deviation, skewness, and kurtosis checking for floor and ceiling effects. The item-rest correlation was used as a measure of homogeneity for polytomous items. For the possible exclusion of items, an item exclusion was taken into account if the *r* ≤.20 (insufficient information to represent the construct) or *r* >.85 (possible overlapping).

#### Internal structure validity

The wide heterogeneity of the DUWAS versions was taken into account to verify its dimensionality. Two paths were taken (a) the evaluation of a confirmatory model to identify the most plausible structure in the target sample and (b) the identification of an exploratory graph model to identify the best community of items based on clustering algorithms. For the confirmatory factor analysis (CFA), diagonal weighted least squares estimator (DWLS) was used with the robust method due to the ordinal nature of the items [71]. The following models were proposed for DUWAS: (a) Model 1 (M1) composed of a unidimensional model; (b) Model 2 (M2) based on the general structure of the WE and WO factors; (c) Model 3 (M3) based on M2 excluding item 5 (identified with an excluding item); and (d) Model 4 (M4) composed of a structure of four correlated factors. To evaluate the fit of the CFA models, the comparative fit index (CFI), Tucker-Lewis index (TLI), root mean square error of approximation (RMSEA), and standardized root mean square (SRMR) were calculated. For the CFI and TLI indices, values between 0.90 and 0.95 are considered acceptable for the goodness of fit of a model [72]. For the RMSEA and SRMR indices, values less than 0.08 are considered acceptable and indices less than 0.05 are good [73].

In addition, the exploratory graph model (EGA) that allows the identification of communities similar to factors in a network structure was evaluated. This type of network is called a psychometric network, the variables are represented as nodes (circles), and the relationships between nodes as edges (lines). Graphical least absolute shrinkage and selection operator (GLASSO) [74] was used to estimate the dependency relationships by identifying a dispersed structure between the variables in network modeling. The walktrap algorithm that can implement random walks between nodes using the Ward method was employed for a better choice of node composition in the communities. The criterion of topological weights of overlap (wTO) was taken into account, values greater than 0.30 indicate high redundancy [75, 76]. The stability of the items was evaluated through the frequency of occurrence of the items in the possible replicated dimension using the Rand index [77, 78].

#### Measurement invariance

Assessing measurement invariance involves testing a series of hierarchically nested models to examine whether the model is stable across two or more groups, so comparisons can be made between them [79]. Comparisons were made between age groups such as youth (18 to 35 years) and adults (> 35 years) through a multigroup confirmatory model (MG-CFA). To compare the models with different progressive restrictions, change criteria were evaluated against models with fewer restrictions through a set of fit indices (CFI, TLI, SRMR, and RMSEA). First, we assessed configural invariance (similar structure across groups), metric invariance (i.e., invariant structure and loading factors across groups), scalar invariance (i.e., similar factor structure, factor loadings and thresholds across groups), and finally strict invariance. (i.e., loading factors, threshodls, and error structure). The *x*^2^ difference was not taken into account in this analysis due to its sensitivity to sample size. For analyses, assessment of ΔCFI was preferred; ΔTLI; ΔSRMR and ΔRMSEA. More liberal criteria were taken into account at initial levels of invariance (i.e., CFI of ≤ 0.02 or RMSEA of ≤ 0.03) [80, 81]. In the case of the evaluation of scalar invariance, a more rigid criterion was maintained ΔCFI < 0.01 or ΔRMSEA < 0.015 [82, 83].

#### Reliability

Internal consistency was evaluated using Cronbach’s alpha (α) and McDonald’s omega (ω). Values between 0.70 and 0.80 are considered acceptable, and values of 0.80 to above, high consistency, and values of 0.95 to above may indicate overlap between items [84, 85]. The bootstrap method was used to obtain the 95% confidence intervals (95% CI) of both reliability coefficients [86]. Corrected and accelerated bootstrap was used to adjust for possible bias and skewness in the bootstrap distribution [87, 88]. In addition, marginal reliability values were added.

#### Concurrent validity

Discriminant validity was determined by the correlation of the DUWAS and UWES scale scores. The correlation was determined by linear correlation Pearson (*r*). The size of the correlation coefficient can indicate a large (*r* >.50), moderate (*r* >.30), or small (*r* >.10) relationship [89].

### Ethics

The Ethics Committee of the Instituto Peruano de Orientación Psicológica – IPOPS (IPOPS-016-2022) approved the study protocol. The DUWAS was administered only under conditions of voluntariness, anonymity, and non-remuneration in people who were previously informed about the aims and purposes of the study. Only participants who fully accepted their participation on a voluntary and non-remunerated basis were counted.

## Results

### Characteristics of the participants

The Spanish version of DUWAS was analyzed and responses were obtained from 446 participants, women were the majority group (60.5%) and the average age was 35.1 years (*SD* = 12.45). 72.9% mentioned being single. The average number of formal work hours was 38.1 (*SD* = 12.6) and 42.8% had a monthly income of less than $270 (see Table 1).

**Table 1:** Socio-demographic characteristics

		*n*	%
*sex*			
	male	176	39.5
	female	270	60.5
*civil status*			
	single	325	72.9
	married	108	24.2
	widower	5	0.4
	divorce	8	2.5
*working hours*			
	stable	271	60.8
	rotary	175	30.2
*live with*	only parents	264	59.2
	independent	134	30
	other family	48	10.8
monthly income*	less than 270$	192	42.8
	270$ a 670$	161	36.1
	671$ a 1071$	54	12.1
	> 1071$	39	9

Note. * monthly income was converted to dollars (1 dollar is equivalent to 3.71 nuevos soles)

### Item analysis

Table 2 shows the mean (*M*) standard deviation (*SD*) and the corrected item-total correlation (ritc) for all DUWAS items. Low scores were identified in item 9, and the highest average of the items was 2.54. Asymmetry and kurtosis were found among acceptable values. The discrimination index was identified between adequate values in most items (ritc = 0.39-0.55).

**Table 2:** Descriptive analysis of the items of the DUWAS scale

items	M	SD	g_1_	g_2_	*r*_itc_	floor	ceiling
1. I seem to be in a hurry and racing against the clock.	2.18	0.90	0.50	-0.43	0.49	22.8	10.3
2. I find myself continuing to work after my co-workers have called it quits.	2.22	0.97	0.38	-0.82	0.49	25.7	12.4
3. It is important to me to work hard even when I don’t enjoy what I’m doing.	2.01	0.95	0.61	-0.57	0.49	35.5	8.7
4. I stay busy and keep many irons in the fire	2.54	0.86	0.14	-0.67	0.55	9.6	14.9
5. I feel that there is something inside me that drives me to work hard.	2.06	0.91	-0.49	-0.61	0.19	7.1	31.8
6. I spend more time working than on socializing with friends, on hobbies, or on leisure activities.	2.49	1.01	0.05	1.09	0.44	19	19.7
7. I feel obliged to work hard, even when it’s not enjoyable.	1.82	0.89	0.83	-0.22	0.50	45	5.4
8. I find myself doing two or three things at one time such as eating and writing a memo while talking on the telephone.	2	0.92	0.58	-0.53	0.50	34.5	7.4
9. I feel guilty when I take time off.	1.34	0.68	2.17	4.49	0.39	75	2.5
10. It is hard for me to relax when I’m not working.	1.54	0.78	1.44	1.52	0.39	60.6	3.3

Note: g1 = skewness; g2 = kurtosis; ritc = item total correlation

### Internal structure validity

**Fig. 2:**
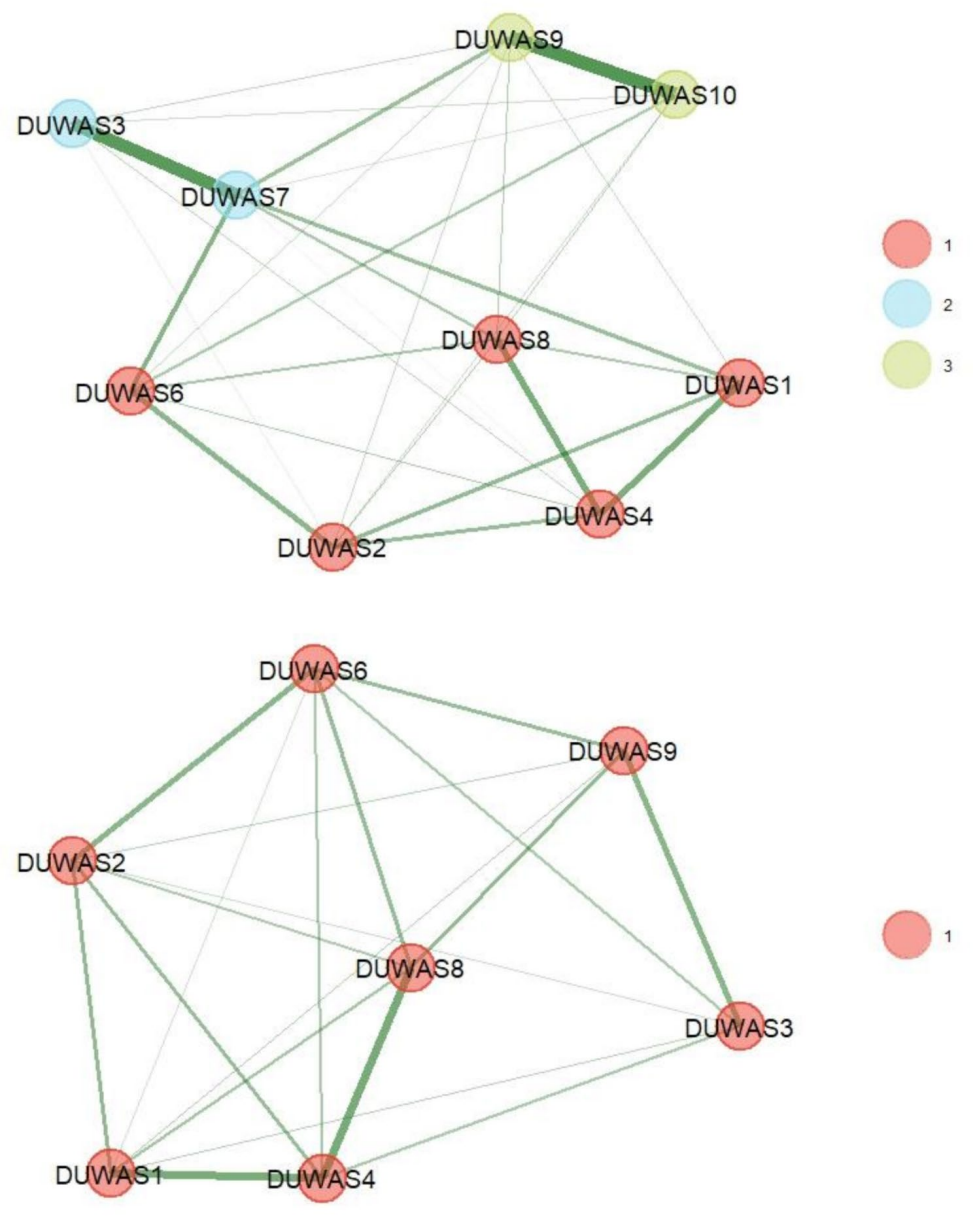
Exploratory graph model for the DUWAS version with and without redundant

**Fig. 3:**
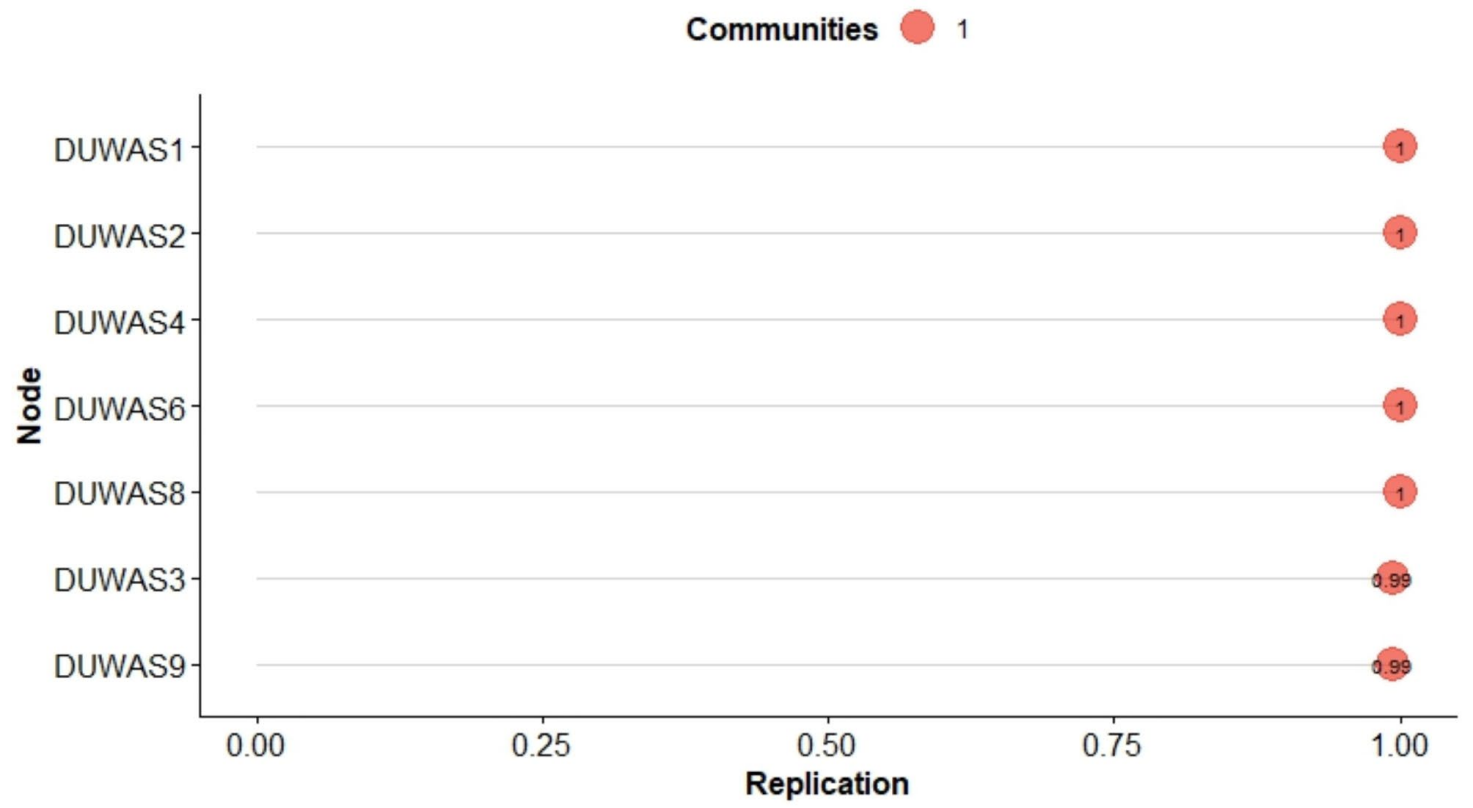
Item stability for models with and without redundant items

In the study, the one-dimensional model and the original model obtained poor fit indices (CFI < 0.90; TLI < 0.90; RMSEA > 0.099) (see Fig. 1). The two-factor model excluding item 5 was partially acceptable, similar to the four-factor model (see Table 3). To verify the possibility of clearer communities of items, the EGA model was used, which identified two pairs of highly redundant items 9<->10 and 3<->7 (wTO > 0.36). Of these pairs of items, only those with greater meaning for addictive behavior were retained and two pairs of network structures were identified (Fig. 2). The stability of the items was clearer in the unidimensional model (see Fig. 3). This model obtained better-fit indices CFI = 0.967; SRMR 0.050; RMSEA = 0.69. In addition, it has acceptable factor loadings in most cases (λ > 0.42).

**Table 3:** Factor analysis for plausible DUWAS models

	x^2^	CFI	TLI	SRMR	RMSEA	IC 90%
M1	488.508 (35)	0.852	0.810	0.089	0.126	0.117–0.136
M2	366.607 (4)	0.891	0.856	0.081	0.110	0.110–0.120
M3	233.432 (26)	0.928	0.900	0.068	0.099	0.088–0.111
M4	242.913 (32)	0.931	0.903	0.067	0.090	0.080–0.101
M5	67.495(14)	0.967	0.950	0.050	0.069	0.053–0.085

Note. CFI = Comparative fit index; TLI = Tucker Lewis Index; SRMR = standardized root residual mean; RMSEA = Root mean standardized error approximation. M1 = one-dimensional model; M2 = WE and WO model; M3 = three-factor model (WE, CO without 5item); M4 = correlated 4-factor model of WE and WO components; M5 = EGA model unidimensionality

### Reliability

The internal consistency of DUWAS was adequate, reporting values of α = 0.735 (95% CI: 0.706 − 0.762) and ω = 0.741 (95% CI: 0.712 − 0.767). Marginal reliability was 0.75. The error of measurement was SEM = 0.22.

### Measurement invariance

For validity comparisons of the models by age and sex, ΔCFI was taken into account; ΔTLI and ΔSRMR were <. 015 relaxing the constraint so that ΔRMSEA were < 0.02 (Table 4). This means that the DUWAS measurement was invariant between the groups by sex. However, for the age group, I only identify as being invariant at the level of indicators (see Table 4).

**Table 4:** Measurement invariance for sex and age variables

Model	x^2^ (df)	CFI	TLI	RMSEA	SRMR	ΔCFI	ΔTLI	ΔRMSEA	ΔSRMR
*Sex*									
Configural	90.933 (28)	0.961	0.942	0.074	0.056	-	-	-	-
Metric	103.186 (35)	0.958	0.950	0.069	0.056	0.003	0.008	0.005	< 0.001
Scalar	95.897 (41)	0.966	0.966	0.057	0.058	0.008	0.016	0.012	0.001
Strict	113.284 (47)	0.959	0.964	0.059	0.059	0.007	0.002	0.002	0.001
*Age*									
Configural	86.379 (28)	0.965	0.948	0.072	0.055	-	-	-	-
Metric	101.304 (35)	0.960	0.952	0.068	0.055	0.005	0.005	0.003	< 0.001
Scalar	94.929 (41)	0.968	0.967	0.057	0.057	0.007	0.015	0.011	0.003
Strict	132.196 (47)	0.949	0.995	0.067	0.057	0.019	0.013	0.010	< 0.001

Note. df = degrees of freedom; CFI = comparative fit index; TLI = Tucker Lewis index; RMSEA = root mean square error of approximation; SRMR = standardized root mean residual; ΔCFI = delta of CFI; ΔTLI = delta TLI; ΔRMSEA = delta RMSEA; ΔSRMR = delta SRMR

### Concurrent validity

The DUWAS measure showed small correlations with work engagement (*r* = -12). In addition, correlations identified with depression, anxiety, and seeking sensation were positive but effect small (*r* <.50). In general, the findings partially show concurrent validity (Table 5).

**Table 5:** Mean scores, standard deviations, correlation coefficients

	1	2	3	4	5
1. Work addiction	-				
2. Engagement	−.12**	-			
3. Depression	0.28**	− 0.44**	-		
4. Anxiety	0.30**	− 0.38**	0.71**	-	
5. Seeking sensation	0.12**	− 0.14**	0.19**	0.13**	-
*M*	14.77	43.20	13.91	10.72	24.36
*SD*	3.92	9.35	4.50	3.95	5.99

*Note*. **p <.001

## Discussion

The unidimensional version of the DUWAS was detected based on the application of the EGA model, which showed adequate psychometric properties. We also identified that this version is reliable and invariant between sex groups and partially with age (i.e. the last level of invariance was not reached). The general DUWAS score was more strongly related to anxiety and depression in the Peruvian population.

The unidimensionality of the DUWAS was confirmed in this study. Although DUWAS was created under a two-dimensional conception (WC and WE), our findings reinforce the idea of unidimensionality from two fronts (a) this addictive behavior is part of a continuum of indicators of behavioral processes of addiction (i.e. not only derived from work excessive) and the cognitive effort (obsession) that determines the repetition of the action of working (i.e. beyond compulsion) [90]. This refers to open behavior, sensations, thoughts, and feelings, which will later determine pathological behavior (repetitive and excessive) within a single factor [37] and (b) The analysis of redundant items helped to identify the overlap more clearly of indicators not explored in previous studies [4, 50, 53]. Despite this, the evidence of unidimensionality is similar and plausible as other addictive behaviors (social media, internet addiction, gambling, etc.). This would indicate that the one-dimensional model of DUWAS is plausible for the population of Peru.

The unidimensional version of DUWAS showed acceptable reliability values and was superior to two-dimensional studies with similar sample sizes [41, 46–50, 53, 55]. A problem widely identified in studies previous was the highly heterogeneous reliability scores, only the dimension WE being the one that presented the most precision [4, 53, 56, 60]. Our study identified acceptable scores based on a unidimensional model that excluded potentially overlapping items, reducing irrelevant model variance. Even though two different reliability coefficients were reported (i.e. α and ω), we identified better coherence between these scores unlike the South African study [53]. The Spanish version is accurate for identifying work addiction score based on seven items.

Measurement invariance with the DUWAS has been poorly explored for sex and age groups. Verification of invariance allows formal comparisons to be made between comparable groups of the measured construct [91]. The professional pressure placed on women by multitasking activities can have an impact on their work performance [3, 47], facilitating greater conditions for WA in this specific group [25, 51]. On the other hand, the age group may perceive WA differently, for example, as an obligation to achieve status and privileges (i.e. adults) or hard work as the action to avoid some punishment (i.e. young people). However, our concerns about invariance for sex and age were not manifested with both groups being validly comparable [92]. Previous studies related to the DUWAS evaluated invariance in cultural groups to verify differences in working conditions [4, 43, 60] and temporal invariance to identify the deterioration of the construct [56]. Despite this, this study shows the way to make valid comparisons at the level of sex and age. Despite the possible structural and social differences between sex and age groups, no apparent differences were identified at the construct level.

The DUWAS scores showed good concurrent validity with psychopathological variables (i.e. anxiety and depression). This is the first time that other psychopathological indicators of anxiety (maintaining the need to work) and depression (escape behavior from work) related to WA have been evaluated. In addition, sensation seeking was included as an immediate reward reinforcer, which is a pattern shared with WA. Work addiction can cause health problems for workers, problems in their work performance, and also in their personal and family lives [38]. This causes the diversion of emotions, cognition, and self-esteem to conclude days of pressure at work, directly related to indicators of anxiety and/or depression [93]. The DUWAS scores are inverse to work engagement [94, 95], which would imply that feelings of ill-being and avoidance behaviors could be related to the work environment [95, 96]. Sensation-seeking is a bimodal trait of goal-oriented (functional) and direct sensation-seeking (dysfunctional) [97, 98]. The low relationship with WA may be due to the potential positive work engagement in boosting opportunities for engagement in activities related to entrepreneurial behavior that are core features of positive work [99].

### Strengths and limitations

We identified some strengths and limitations in our study. First, this study identifies new valid comparisons between sex and age variables important in the course of this addictive behavior that had not been previously explored. A method based on the identification of communities (items) was used to capture redundant items. Self-reports were used that could have recall bias. To try to control this bias, questions aimed at work addiction were carefully included. The measurement of invariance did not take other important variables in the work context (e.g. effective work hours, other work groups), other addictive behaviors (i.e. energy drinks, caffeine), and other variables were not added to health (e.g. sleep problems, stomach disorders, etc.). Despite these limitations, our study provides new information that supports the unidimensionality of work addiction in Spanish-speaking contexts.

## Conclusion

Our study concludes that the DUWAS-7 version shows evidence of validity and reliability for the one-factor model. In addition, the DUWAS-7 was invariant, which implies that valid comparisons can be made between groups (i.e. sex and, partially, age). Finally, we recommend the use of the DUWAS-7 version for the general population of Peru.

## Electronic supplementary material

Below is the link to the electronic supplementary material.

Supplementary Material 1

## Data Availability

The database can be accessed by requesting it from the authors.
