# Supplementary material for: Responding to old problems in the Dutch work addiction scale: a psychometric approach in a Peruvian sample
Source: BMC Psychol. 2025 Feb 22;13:155. doi: 10.1186/s40359-025-02437-1 (PMC11847342; doi:10.1186/s40359-025-02437-1)
Supplement: Supplementary file 1 — Supplementary Material 1 [file 40359_2025_2437_MOESM1_ESM.docx]

## Supplemental

Table S1

Psychometric measurements of DUWAS

| author(s) | sample | analysis | version | model fit | factors |
| --- | --- | --- | --- | --- | --- |
| Schaufli (2009) | Deutchland (n=3797) Japan ( n = 1655) | EFA (varimax, principal components). | 10 ítems: Work Excessively (5 items), Work Compulsive (5 items). | not apply | 2 |
| Líbano et al. (2010) | Netherlands (n= 2164) Spain (n= 550) | CFA Function: Estimated method non indicated | 10 ítems: Work Excessively (5 items), Work Compulsive (5 items). | Dutch: χ2= 471.68, df= 32, GFI= .96, RMSEA= .08, TLI= .90, CFI= .93. Spain: χ2= 123.90, df= 32, GFI= .96, RMSEA= .07, TLI= .93, CFI= .95. | 2 |
| Falco et al. (2012) | Italy (n= 243) | CFA  Function: ML | 10 items: Work Excessively (6 items), Work Compulsive (4 ítems) | χ2= 164.52, df= 33), RMSEA= .089; CFI= 97, NNFI= .96, SRMR= .044 | 2 |
| Molino et al. (2012) | Italy (n= 853) | EFA and CFA  Function: ML | 5 items: Work Excessively (5 items) | χ2= 11.39, df= 4, NNFI= 99, CFI= .99, RMSEA= .05 | 1 |
| Andreassen et al. (2012) | Norwegian (n= 661) | CFA and EFA Function: Principal component analysis | 10 items: Work Excessively (5 items), Work Compulsive (5 items). | (Final fit non indicated in EFA model) | 2 |
| Littman-Ovadia et al. (2014) | Hebrew (n= 351) | CFA Function: ML Robust with Satorra-Bentler correction) | 10 items: Work Excessively (5 items), Work Compulsive (5 items). | χ2= 139.099, df= 34, SRMR= .071, CFI= .907, RMSEA= .096. | 2 |
| Balducci et al. (2015) | Italian (n= 1027) Deutch (n= 7523) | CFA Function: ML | 10 items: Work Excessively (5 items) and Work Compulsive (5 items). | Italian: χ2= 188.229, df= 34, SRMR= .041, CFI= .967, NCI= .928, RMSEA= .068 (058, .077).  Deutch: χ2= 1,576.636, df= 34, SRMR= .055, CFI= .957, NCI= .903, RMSEA= .079 (076, .082). | 2 |
| Rantanen et al. (2015) | Netherlands (n= 9010) Finland (n= 4567) | CFA Function: ML Robust with Satorra-Bentler correction | 10 items: Work Excessively (working frantically= 3 ítems, working long hours= 2 ítems) and Work Compulsive (obsessive work drive= 3 items, unease if not working= 2 items). | Dutch: χ2(30)= 424.31, RMSEA=.05, SRMR=.03, CFI=.96, TLI=.94  Finland: χ2(30)= 218.81, RMSEA= .05, SRMR=.03, CFI= .97, TLI= .96 | 4 |
| Sandrin & Gillet (2016) | French group 1 (n= 411) French group 2 (n= 254) | CFA Function: ML | 10 items: Work Excessively (5 items), Work Compulsive (5 items). | French group 1: χ2= 121.55, df= 3.68, GFI= .95, CFI= .90, IFI= .90, RMSEA= .08 (.07, .10).  French group 2: χ2= 39.38, df= 1.19, GFI= .97, CFI= .99, IFI= .99, RMSEA= .03 (.00, .06). | 2 |
| Mir et al. (2016) | Pakistani (n= 317) | CFA Function: ML | 8 items: Work Excessively (5 items), Work Compulsive (3 items). Remove items 9 and 10. | χ2= 49.49, df= 19, GFI= .96, AGFI= .93, IFI= .92, CFI= .92, RMSEA= .07 | 2 |
| Azevedo & Telles (2017) | Brazil (n= 1108) | EFA Function: Principal Component Analysis | 10 items: Work Excessively (5 items), Work Compulsive (5 items). | not apply | 2 |
| Omar et al. (2018) | Argentine (n= 459) | EFA and CFA Function: ML Robust with Satorra-Bentler correction | 10 items: Work Excessively (5 items), Work Compulsive (5 items). | χ2= 2.35, GFI= .90, CFI= .96, RMSEA= .03 (.04, .05), AIC= 150.31. | 2 |
| Souza et al. (2018) | Brasileira (n= 571) | CFA Function: WLSMV | 10 items: Work Excessively (5 items), Work Compulsive (5 items). | χ2= 169.68, gl (34), CFI= .93, TLI= .91. | 2 |
| Borges et al. (2021) | Portuguese (n= 1030) | CFA Function: ML | 9 items: Work Excessively (5 items), Work Compulsive (4 items). Remove item 9. | χ2= 211.627, gl= 31, RMR= .032, CFI= .932, RMSEA= .075 (.066, .085). | 2 |
| Beer et al. (2022) | South African (n= 345) | CFA Function: WLSMV | 9 items: Work Excessively (5 items), Work Compulsive (4 items). Remove item 3 (Item 5 in original version) | CFI= 918, TLI= .911, RMSEA= .064 (.060, .068), SRMR= .069. | 2 |

Note. EFA = exploratory factor analysis; CFA = confirmatory factor analysis; ML = maximum likelihood; WLSMV = weighted least squares mean and variance adjusted; CFI = Comparative fit index; TLI = Tucker Lewis Index; SRMR = standardized root residual mean; RMSEA = Root mean standardized error approximation; GFI = Goodness- of-fit index; AGFI = Adjust goodness-of-fit index; NNFI = Non-normed fit index; NFI = Normed fit index; IFI = Incremental Fit Index.
